# West Nile virus: characterization and diagnostic applications of monoclonal antibodies

**DOI:** 10.1186/1743-422X-9-81

**Published:** 2012-04-13

**Authors:** Davide Lelli, Ana Moreno, Emiliana Brocchi, Enrica Sozzi, Lorenzo Capucci, Elena Canelli, Ilaria Barbieri, Herve Zeller, Paolo Cordioli

**Affiliations:** 1 Istituto Zooprofilattico Sperimentale della Lombardia e dell'Emilia Romagna, Via Bianchi 9, 25124 Brescia, Italy

**Keywords:** West Nile virus, Monoclonal antibody, Epitope

## Abstract

**Background:**

Diagnosis of West Nile virus (WNV) infections is often difficult due to the extensive antigenic cross-reactivity among flaviviruses, especially in geographic regions where two or more of these viruses are present causing sequential infections. The purpose of this study was to characterize a panel of monoclonal antibodies (MAbs) produced against WNV to verify their applicability in WNV diagnosis and in mapping epitope targets of neutralizing MAbs.

**Methods:**

Six MAbs were produced and characterized by isotyping, virus-neutralization, western blotting and MAb-epitope competition. The MAb reactivity against various WNVs belonging to lineage 1 and 2 and other related flaviviruses was also evaluated. The molecular basis of epitopes recognized by neutralizing MAbs was defined through the selection and sequencing of MAb escape mutants. Competitive binding assays between MAbs and experimental equine and chicken sera were designed to identify specific MAb reaction to epitopes with high immunogenicity.

**Results:**

All MAbs showed stronger reactivity with all WNVs tested and good competition for antigen binding in ELISA tests with WNV-positive equine and chicken sera. Four MAbs (3B2, 3D6, 4D3, 1C3) resulted specific for WNV, while two MAbs (2A8, 4G9) showed cross-reaction with Usutu virus. Three MAbs (3B2, 3D6, 4D3) showed neutralizing activity. Sequence analysis of 3B2 and 3D6 escape mutants showed an amino acid change at E307 (Lys → Glu) in the E protein gene, whereas 4D3 variants identified mutations encoding amino acid changed at E276 (Ser → Ile) or E278 (Thr → Ile). 3B2 and 3D6 mapped to a region on the lateral surface of domain III of E protein, which is known to be a specific and strong neutralizing epitope for WNV, while MAb 4D3 recognized a novel specific neutralizing epitope on domain II of E protein that has not previously been described with WNV MAbs.

**Conclusions:**

MAbs generated in this study can be applied to various analytical methods for virological and serological WNV diagnosis. A novel WNV-specific and neutralizing MAb (4D3) directed against the unknown epitope on domain II of E protein can be useful to better understand the role of E protein epitopes involved in the mechanism of WNV neutralization.

## Background

West Nile virus is an arbovirus member of the Japanese Encephalitis virus (JEV) serocomplex of the genus *Flavivirus *of the *Flaviviridae *family. WNV infection is one of the most widespread arboviral infections and can cause encephalitis in humans. Its transmission cycle involves mosquito-vectors (mainly *Culex spp*.) and birds as amplifying reservoirs, but a wide variety of vertebrate species, including reptiles, amphibians and mammals, such as equines and humans, are also susceptible to infection [1].

The WNV genome is made up of a single stranded positive-sense RNA molecule that encodes three structural proteins (capsid (C); pre-membrane (prM); and envelope (E)) and seven non-structural proteins (NS1, NS2A, NS2B, NS3, NS4A, NS4B, NS5) [2]. The envelope E protein is the major surface protein of flaviviruses and the primary immunogen that plays a central role in virus attachment and entry into a cell via membrane fusion [3]. Crystallographic analysis reveals that the E glycoprotein of flaviviruses folds into three distinct structural domains (I, II and III) [4-6]. In particular, domain III of WNV E protein (DIII) is the putative receptor-binding domain and is an important target for neutralizing antibodies and in vivo protection [7-11]. The recent outbreaks of West Nile Disease in humans and horses in Europe and the spread of the virus from North through South America during the last decade suggest that the epidemiology of this infection is evolving. In the Mediterranean basin, outbreaks of WNV infection in recent years have been reported in France (2004 and 2006), Italy (2008, 2009) Morocco (2010), Spain (2010) and Greece [12]. WNV was previously considered an exotic agent, while it is now regarded as an emerging problem for both human and veterinary public health. These outbreaks have stimulated research into virus detection and characterization, underlining the need for rapid assays. Although many methods have been developed for WNV diagnosis, it is commonly difficult due to the extensive antigenic cross-reactivity among flaviviruses, especially in geographic areas where two or more of these viruses are present causing sequential infections [13]. It has recently been shown that WNV and Usutu virus (USUV) have similar transmission cycles, with overlapping geographic distributions [14,15]. In this context, MAbs having strong and specific reactivity to WNV antigens are the most suitable choice for the development of standardized diagnostic tools.

The purpose of this study was to characterize a panel of monoclonal antibodies produced against WNV to verify their applicability in WNV diagnosis and in mapping epitope targets of neutralizing MAbs.

The results suggest the applicability of these MAbs to various analytical methods for WNV diagnosis allowing the characterization of a novel WNV-specific and neutralizing epitope located on DII of E protein that has not previously been described with WNV MAbs.

## Results

### Selection and characterization of monoclonal antibodies

During the screening phase of hybridomas, several MAbs that were reactive to the WNV antigen were obtained. Six hybridomas (3B2, 3D6, 1C3, 4D3, 2A8, 4G9) showing a strong signal with indirect ELISA and immunofluorescence (IF) against homologous WNV were selected and cloned by limiting dilution to ensure monoclonality and stability. Positive clones that secreted a high titer of selected antibodies were further identified and MAbs were efficiently purified by protein-A chromatography and conjugated with horseradish peroxidase (HRP). The general characteristics of selected anti-WNV MAbs are summarized in Table 1.

**Table 1:** Properties of selected anti-WNV MAbs

MAbs	IPMA			VNT		WB	Isotype
			
	WNVs	USUVs	TBEV	WNV Eg101	WNV B956		
**3B2**	+	-	-	12800	12800	52 Kd	IgG2a

**3D6**	+	-	-	12800	12800	52 Kd	IgG2a

**4D3**	+	-	-	1600	1600	-	IgG2b

**1C3**	+	-	-	< 20	< 20	-	IgG2a

**2A8**	+	+	-	< 20	< 20	-	IgG2a

**4G9**	+	+	-	< 20	< 20	-	IgG2a

IPMA: Detection of WNVs, USUVs and TBEV infected Vero cells by immunoperoxidase assay. WNVs used are: Eg101, B956 ATCC, NY99 ATCC, Central African Republic 1967, 203204/08, 225677/09, 208659/09, 204913/09. USUVs used are: SAAR 1776, Vienna 2001-blackbird and 200092/2010. TBEV used is 103457/09. + indicate reactivity against all viral strains tested, - indicate absence of reactivity with all viral strains.

VNT: Virus neutralization against WNV Eg101 and WNV B956. Neutralizing titers are calculated as reciprocal of MAb dilution producing 100% reduction of CPE. MAbs were used as ascites fluid.

All six MAbs showed good reactivity with Immunoperoxidase Monolayer Assays (IPMA) staining on all the eight WNVs tested. Four MAbs 3B2, 3D6, 4D3 and 1C3 resulted WNV-specific without any cross-reaction to USUVs and Tick-borne encephalitis virus (TBEV), and three of these (3B2, 3D6, 4D3) showed neutralizing activity at the same titers against both the homologous WNV Eg101 and the prototype lineage 2 WNV B956. MAbs 2A8 and 4G9 showed cross-reaction with the two USUVs tested, but not with TBEV. No neutralizing activity was observed. The heavy chain subclasses were determined as IgG2a for all MAbs with the exception of 4D3, which was determined as IgG2b. The light chains of all of these were kappa isotype.

**Figure 1:**
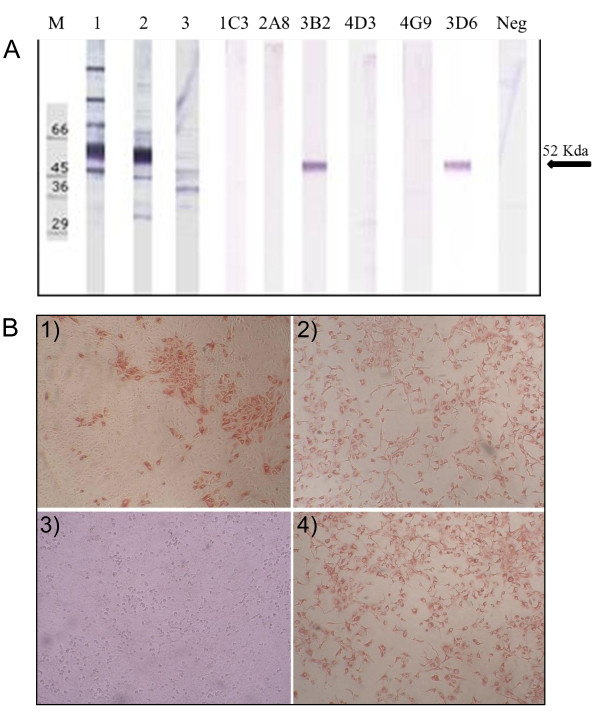
**(A) Western blot analysis**. Reactivity of six anti-WNV MAbs with denatured antigen. A MAb against influenza virus was used as negative control. M: protein molecular weight markers. Lane 1: sera from WNV infected horse. Lane 2: sera from WNV vaccinated horse. Lane 3: sera from WNV negative horse. **(B) Detection of WNV and USUV-infected cells using IPMA (10X)**. WNV-infected Vero cells stained with MAb 3B2 (1) and 2A8 (2). USUV-infected VERO cells stained with MAb 3B2 (3) and 2A8 (4).

Two MAbs (3B2, 3D6) resulted positive at western blotting (WB), revealing a band with a molecular weight of 52Kd corresponding to E protein. The remaining MAbs did not recognize denatured WNV antigen at WB, suggesting that they are all directed to conformational-dependent epitopes (Figure 1A). Figure 1B shows IPMA assays performed with WNV, USUV and MAbs 3B2 and 2A8.

None of the MAbs showed any cross-reaction against Dengue virus serotype 1, 2, 3 and 4 (DENV 1, 2, 3, 4), Yellow fever virus (YFV), TBEV and Japanese Encephalitis virus (JEV) by indirect ELISA.

### Competition binding ELISAs for MAb epitope studies

To determine if the selected MAbs would bind to overlapping epitopes, we tested MAbs using competitive binding ELISAs. For these studies, the saturating concentrations of unlabeled MAbs were incubated with partially purified WNV antigen bound to the ELISA plates, after which an HRP-conjugated "competitor" MAb was added. If preincubation of the antigen with an unlabeled MAb blocked subsequent binding of a conjugated MAb, we concluded that MAbs bind to similar or at least overlapping epitopes. The optimal dilution of each HRP-conjugated MAb was determined by testing serial two-fold dilution in WNV-coated ELISA plates and selecting this as the working dilution to generate an optical density of around 1.5. The selected working dilution was 1/8000 for MAb 3B2, 1/16000 for MAbs 3D6, 2A8, 4G9 and 1/4000 for 4D3, 1 C3. The results of competitive ELISAs are reported in Table 2.

**Table 2:** Results of competition binding ELISAs for MAb epitope studies

	Unlabelled MAbs	HRP-conjugated MAbs					
		
		3B2 HRP	3D6 HRP	1C3 HRP	4D3 HRP	2A8 HRP	4G9 HRP
Group 1	3B2	4	6	85	64	90	80
	
	3D6	4	5	86	65	87	75

Group 2	1C3	87	95	7	60	75	60

Group 3	4D3	85	93	91	5	88	72

Group 4	2A8	95	100	84	65	4	4
	
	4G9	92	100	84	63	4	3

no MAb		100	100	100	100	100	100

Result expressed as percent binding of HRP-conjugated MAbs. The amount of binding obtained in the absence of unlabeled antibody was set at 100% for each HRP conjugated MAb. First column indicates the group assignments of MAbs based on competition binding assay. MAbs binding to overlapping epitopes are grouped together, while non-competing MAbs are grouped individually.

HRP-conjugated 3B2 inhibited the binding of 3D6 to the antigen and vice versa, showing that 3B2 and 3D6 recognized overlapping epitopes. 4D3 and 1 C3 did not significantly compete with any of the other MAbs tested in this assay, which meant that 4D3 and 1 C3 recognized two distinct epitopes different from 3B2, 3D6, 2A8 and 4 G9. HRP-conjugated 2A8 inhibited the binding of 4G9 to the antigen and vice versa, indicating that 2A8 and 4G9 recognized overlapping epitopes.

One-way partial competition observed for some MAbs, as for 4D3 HRP, could be due to antibody-induced conformational changes in E protein upon antibody binding or steric hindrance. MAbs were thus categorized into four groups. MAbs binding to the same epitopes or overlapping epitopes were put into the same group, while non-competing MAbs were grouped individually (Table 2). Group one consisted of MAbs 3B2 and 3D6, group two included MAb 1 C3, group three included MAb 4D3 and group four consisted of MAbs 2A8 and 4G9.

### Antigen capture ELISA (AC-ELISA)

**Figure 2:**
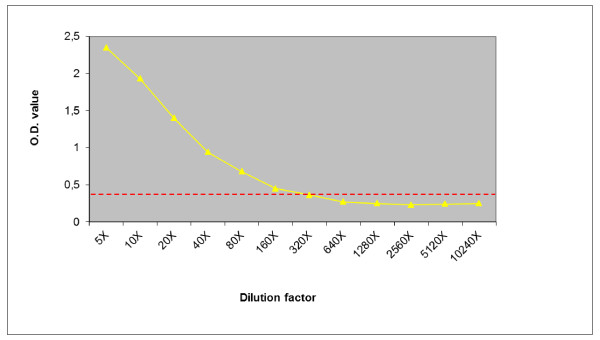
**Sensitivity of AC-ELISA using MAb 3B2 and WNV cell culture supernatant**. Detection limit from 200- to 300-fold dilution of cell culture supernatant which was 10^3,8^TCID_50_/0,1 ml. The broken line indicates the absorbance ratio cut-off value.

All possible combinations of trapping and conjugated MAbs were evaluated to establish a sensitive AC-ELISA for WNV detection. Each pair of MAbs was able to detect all WNVs when used in sandwich ELISA without significant differences in sensitivity. No reactivity to TBEV was observed while pairs of MAbs formed by 2A8 and 4 G9 allowed the detection of USUVs (data not shown). Good WNV-specificity combined with the highest signal was obtained using 3B2 both as capture antibody and HRP-conjugated as detector antibody. To determine the detection limit of AC-ELISA, serial dilutions of WNV Eg101 culture supernatant (10^5.1 ^TCID_50_/100 μl) were used to construct the binding curve (Figure 2). An uninfected cell culture supernatant was used as negative control. According to the cut-off threshold, calculated as the mean of optical density (OD) of negative control plus two standard deviations, the detection limit for the WNV AC-ELISAs was deduced to be 10 ^3.8 ^TCID_50_/100 μl.

### Competitive binding assays with experimental sera

This experiment was designed to evaluate the capability of known experimental sera to inhibit the binding of anti-WNV MAbs to the antigen and then to verify the possibility of using them in competitive ELISA tests for serological diagnosis in various animal species.

Sera from SPF chickens experimentally infected with WNV and equine sera immunized using inactivated vaccine were tested in competitive ELISAs with each HRP-conjugate MAb at a predetermined dilution. The results, expressed as percent inhibition of MAb binding by competition with serum antibodies, are shown in Table 3. Good competition for antigen binding between all MAbs and sample sera collected from vaccinated horse, as well as infected chicken, was observed.

**Table 3:** Percent inhibition of WNV-reactive MAb binding with competitive ELISA using sera from WNV-infected chicken and WNV-vaccinated horse during period between infection and serum collection

	Days post-infection/vaccination	WNV VN titer	% Inhibition of MAb binding					
		
			3B2	3D6	4D3	1C3	2A8	4G9
Chicken	0	< 5	0	4.7 ± 2.1	4.7 ± 1.5	8 ± 2	7 ± 1.2	4.3 ± 1.5
	
	7	10	56.7 ± 2.1	76.7 ± 2.1	43.3 ± 2.5	79 ± 4.6	75.7 ± 1.5	75 ± 3.5
	
	15	20	77 ± 1	80.3 ± 2.1	78.7 ± 2.5	86.7 ± 1.2	87 ± 2.6	91.7 ± 1.5
	
	24	40	81 ± 2.6	80 ± 1.7	83.7 ± 1.5	89.3 ± 1.2	89 ± 1	94.3 ± 0.6
	
	42	160	92.7 ± 1.5	95.3 ± 0.6	91 ± 1	93.7 ± 1.2	95.7 ± 1.2	94.3 ± 1.5

Horse	0	< 5	28.7 ± 2.1	15.7 ± 2.1	47.3 ± 2.5	19 ± 1	23.7 ± 1.5	23.3 ± 2.5
	
	15	< 5	71 ± 2	42.3 ± 1.2	73 ± 1	42.7 ± 0.6	44 ± 1	64.3 ± 1.5
	
	21	10	81.7 ± 1.5	65 ± 1	74.7 ± 0.6	69.3 ± 3.5	65.7 ± 1.5	80.3 ± 2.1
	
	28	20	88.7 ± 0.6	71.7 ± 1.5	85.7 ± 1.5	78.7 ± 1.2	77 ± 2	88.3 ± 0.6
	
	31 (2°v)	80	95.7 ± 0.6	92.7 ± 1.5	91.3 ± 1.2	91.7 ± 1.5	93.3 ± 3.1	95 ± 1

MAbs were tested in triplicate. All sera were diluted 5 fold.

Values are mean ± standard deviations.

### Escape mutant analysis for MAb epitope mapping

To identify residues that might define epitopes recognized by the neutralizing monoclonal antibodies, potential neutralization-resistant escape mutants (EM) were selected by growing WNV Eg101 in Vero cells in the presence of MAbs 3B2, 3D6 and 4D3.

**Figure 3:**
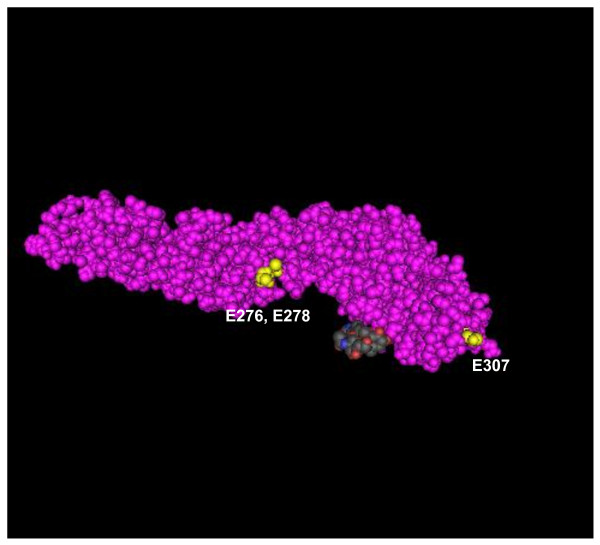
**3D macromolecular structure of WNV E protein based on the crystal structure available in Molecular modelling database (PDB: 2I69-A) visualized by Cn3D version 4.3**. Mutated amino acid residues important for MAbs binding 3B2, 3D6 (E307) and 4D3 (E276, E278) are evidenced in yellow.

Four or five neutralization-resistant escape mutants were identified for each MAb. Virus neutralization assays showed that 3B2 and 3D6 were unable to neutralize both EM3B2 and EM3D6, but retained strong neutralizing activity against EM4D3. On the contrary, MAb 4D3 was unable to neutralize EM4D3, but retained strong neutralizing activity against both EM3B2 and EM3D6. Comparative sequence analysis of viral genome corresponding to E genes of escape mutants and wild-type WNV Eg101 was performed. A similar single-nucleotide mutation was identified on the E-protein genes of both EM3B2 and EM3D6 at residue 919 (AAG → GAG) encoding an amino acid change at E307 (Lys → Glu). Various nucleotide mutations were identified on the E-protein gene of EM4D3 at residue 827 (AGC → ATC) encoding an amino acid change at E276 (Ser → Ile) or at residue 833 (ACT → ATT) encoding an amino acid change at E278 (Thr → Ile). Tridimensional macromolecular structure of E protein showing these amino acid residues are reported in Figure 3. None of these mutations was associated with measurable differences for growth in cell cultures.

Based on the sequence of in vitro neutralization escape variants, MAb 3B2 and 3D6 map to a region on the lateral surface of DIII, which has been previously described as an important neutralizing epitope for WNV [7,16], while MAb 4D3 appears to recognize a novel neutralizing epitope on DII that has not previously been described with WNV MAbs.

## Discussion

West Nile virus is not only a human pathogen, but is also a major veterinary pathogen. One of the principal factors preventing the development of clinical disease due to WNV infection is the presence of protective neutralizing antibodies. However, despite much progress, many unanswered questions remain concerning WNV neutralization mechanisms.

The majority of potent murine neutralizing MAbs described to date against WNV bind to domain III, more specifically to an epitope on the lateral surface of DIII recognized by specific antibodies with the strongest neutralizing activity in vitro and in vivo [7,9,10,16,17]. In this study, six monoclonal antibodies produced against WNV lineage 1 were characterized and their applicability in laboratory diagnosis was assessed. Furthermore, through selection of neutralizing-MAb escape mutants, we defined the molecular basis of two epitopes recognized by our neutralizing MAbs.

We showed that all six MAbs (3B2, 3D6, 4D3, 1C3, 2A8, 4G9) had strong reactivity in IPMA for all the eight WNVs tested, including reference strains belonging to lineages 1 and 2 as well as field strains isolated during the Italian epidemics. This finding suggests that all MAbs recognized conserved regions among WNVs. We also showed the cross-reactivity of 2A8 and 4G9, which recognize the overlapping epitopes shared between WNV and USUV while no MAbs exhibited any cross-reactions with other related flaviviruses tested. Considering that WNV, USUV and TBEV are the flaviviruses currently circulating in Europe, a good knowledge of MAb reactivity against them is very important for their implementation as diagnostic tools.

MAbs 3B2, 3D6 and 4D3 neutralized lineage 1 and lineage 2 WNV. Moreover, they neither neutralized nor recognized USUV and TBEV. MAb-binding sites of neutralizing MAbs have been characterized by the selection and sequencing of MAb escape mutants. We showed that 3B2 and 3D6 recognized the same epitopes located on the distal lateral surface of DIII. Specifically, sequence analysis of 3B2 and 3D6 escape variants showed a single-nucleotide mutation in the E protein gene that encodes an amino acid change at E307 (Lys → Glu). This residue defines an epitope that has previously been described as an important epitope recognized by WNV-specific antibodies with the strongest neutralizing activity [7,9,10,16,17]. Recent studies report that recombinant DIII peptides containing virus-specific epitopes could be used for specific serological diagnosis of flaviviral infections [18-20]. Considering the extensive antigenic cross-reactivity among flaviviruses, we speculated that joining recombinant DIII peptides to anti-DIII MAbs such as 3B2 and 3D6 for the development of serological tests for WNV diagnosis could provide good results as regards specificity. Additionally, Oliphant et al. (2005) showed that MAbs against WNV-DIII have therapeutic potential against this virus, while DIII E peptides could be used as subunit flavivirus vaccines [18,21-23].

Numerous studies on neutralizing monoclonal antibodies outside DIII have been reported [24-27]. We saw that MAb 4D3 recognized a neutralizing epitope on domain II that is a JEV complex epitope, already seen in other studies [28] but which has not been previously described in WNV. Nucleotide mutations were identified on the E gene of 4D3 variants encoding amino-acid changes at E276 (Ser → Ile) or E278 (Thr → Ile). The WNV inhibiting role of this specific neutralizing epitope located on DII needs further investigation to verify its role as a candidate for the development of diagnostic tools, as well as for use as an antiviral vaccine and for therapeutics. The mechanisms of neutralization and the role of the different epitopes of the WNV envelope protein have not been fully understood. According to Sanchez et al. [29], the neutralizing response to WNV generated in infected horses is both variable and polyclonal in nature, with epitopes both within and outside DIII playing important roles. The same study reports that in some horses that were naturally infected with WNV, antibodies directed against DIII epitopes were not consistently detected, and DII may be an important target for neutralizing antibodies in these animals. The anti-domain II MAb 4D3 could be used in competitive tests, in parallel to MAbs directed against DIII, to reveal WNV-neutralizing antibodies in animal species. This is supported by our results obtained with competitive ELISAs designed using experimental horse and chicken sera and HRP-conjugated MAbs. Good competition for antigen binding between MAb and sample sera from vaccinated horse, as well as infected chicken, was observed. Additionally, good competition was also observed in competitive ELISAs between 3B2, 3D6 and infected horse sera collected during the WNV Italian epidemics (data not shown). These results also indicate that all MAbs herein described react to epitopes with high immunogenicity and that they could be used for the development of competitive tests for the detection of different types of anti-WNV antibodies in animal species, e.g. WNV-specific and neutralizing antibodies directed to DIII (MAbs 3B2 and 3D6) or DII (MAb 4D3), WNV-specific non-neutralizing antibodies (Mab 1C3) or flavivirus cross-reactive non-neutralizing antibodies (MAbs 2A8 and 4 G9).

The availability of MAbs with strong reactivity to WNV is also a crucial component for AC-ELISA development usable for large-scale screening in surveillance programs. Various MAb combinations were evaluated for WNV detection using AC-ELISA. It was found that a combination of 3B2 as catcher and tracer MAb gave the highest signal combined with good specificity. The detection limit of 10^3.8^TCID_50_/100 μl for WNV-infected cell culture supernatant obtained in the present study is comparable with that found in previously published AC-ELISA [30]. Considering that AC-ELISAs performed with pairs of MAbs made up of 2A8 and 4G9 allowed the detection of USUVs, these tests could be useful for viral detection in pathological specimens or cell cultures in areas where WNV and USUV spread concomitantly.

## Conclusions

Six MAbs against WNV were produced and characterized. The results suggest the applicability of these MAbs to various analytical methods for antigen or antibody detection in areas such as the Mediterranean basin, where there is contemporary circulation of WNV, USUV and TBEV. Additionally, in this study the novel WNV-specific neutralizing MAb 4D3 directed against the unknown epitope on domain II of the E protein of WNV was generated and characterized.

## Materials and methods

### Viruses

The virus used in this study as an immunogen for MAb production was the WNV reference strain Eg101.

Other WNVs used in this study included: B956 ATCC, NY99 ATCC, Central African Republic 1967, 203204/08 isolated from magpies (*Pica pica*) during the 2008 Italian epidemic and three other Italian field strains (225677/09, 208659/09, 204913/09) isolated in our lab in 2009 from a jay (*Garrulus glandarius*), a horse and mosquitoes (*Culex pipiens*) respectively. Other flaviviruses used included three strains of USUV, reference strain SAAR 1776, Vienna 2001-blackbird and 200092/2010 (Italian field strain isolated in our lab from a blackbird), and TBEV strain 103457/2009 (Western TBEV group) isolated in our lab from the brain of dog that had been in an endemic area (Hungary) for a short period in April 2009. Virus stocks were prepared in Vero cells that were cultured at 37°C with 5% CO_2 _in minimum essential medium (MEM) containing 10% of fetal serum bovine, 0.1 mM of nonessential amino acids, 0.15% sodium bicarbonate, 2 mM _L_-glutamine, penicillin (100 U/ml) and streptomycin (100 μg/ml). Virus titers were determined by endpoint assays in Vero cells.

### Antigen preparation

For its use as an immunogen for MAb production in Balb/c mice and for the preparation of ELISA coating antigen, WNV Eg101 was partially purified as follows. WNV was grown on a preformed monolayer of Vero cells and incubated at 37°C until the cytopathic effect (CPE) is complete. After a freeze-thaw step, virus inactivation was carried out by the addition of 0.05% (v/v) Betapropiolactone to the culture fluids, followed by incubation at 37°C for 2 h plus overnight incubation at 4°C. Virus inactivation in the culture fluid was verified following three blind passages in Vero cell monolayers without CPE development. Inactivated supernatant fluid was clarified by centrifugation at 4000 rpm for 30 min, added to 8% of PEG 6000 and NaCl 0.5 M then placed overnight at 4°C in agitation. The suspension was centrifuged at 5000 rpm for 30 min and the pellet was re-suspended in phosphate-buffer saline solution pH 7.4 (PBS) at 20X concentration compared to the initial volume. Following further clarification by centrifugation (5000 rpm for 30 min), the viral suspension was purified by ultracentrifugation at 35000 rpm for 2 h (rotor TST41 Kontron) through a 25% (w/w) sucrose cushion and the pellet was re-suspended in PBS. This concentrated antigen was kept at -70°C until use.

### Experimental sera production

#### Immune chicken sera

Five SPF chickens were used. Three of these (positive control) were experimentally infected intramuscularly with 1 ml containing 10^6.5^TCID_50 _of WNV Eg101 grown on BHK21, while the remaining two chickens (negative control) were injected intramuscularly with a cell suspension of BHK21. Blood samples were taken 7, 15, 24, 42 days post-infection.

#### Immune horse sera

Two ponies tested seronegative for WNV using a virus neutralization test were vaccinated intramuscularly with 2 doses of commercial inactivated vaccine. Thirty-five days after the first vaccination, a second immunization with two additional doses was performed. Blood samples were taken 15, 21, 28 days after the first vaccination and 31 days after the second vaccination.

Animal care and all procedures were performed in accordance with guidelines and regulations of the Italian animal protection laws and under EC policy related issues (D.L. 116/1992 amending Dir. CEE n. 609/86).

### Monoclonal antibody production

Two Balb/c mice were primed subcutaneously with 50 μg of Betapropiolactone-inactivated WNV with complete Freund's adjuvant and boosted intraperitoneally with the same antigen in PBS (phosphate-buffered saline pH 7.4) once or twice at one month intervals of one month. Three days after the last boost, the mice were sacrificed and hybridomas were generated following fusion of splenocytes with NS0 myeloma cells and selected cultures were grown following the standard procedure [31]. Hybridomas were screened for secretion of desired antibodies with indirect ELISA using the homologous WNV and IF with infected and non-infected Vero cells. Positive hybridoma cells were cloned using limiting dilution to obtain antibodies from a single cell. Hybridoma culture supernatants or ascitic fluids harvested after in vivo culture of hybridoma were used as a MAb source. MAbs were purified from ascites using affinity chromatography through protein A Sepharose (Pharmacia, Milan, Italy) in the presence of 3 M Nacl and 1.5 M glycine pH 8.9 [32], and conjugated with HRP using a modified form of the method described by Tjissen and Kurstak 1984 [33]. The immunoglobulin subclass was determined using a Mouse Immunoglobulin Isotyping ELISA kit (BD Pharmingen) following the manufacturer's instructions.

### Immunofluorescence (IF)

Confluent monolayers of Vero cells grown in 96-well microplates were infected with 100TCID_50 _of WNV Eg101. After incubation for 48/72 hours at 37°C, or following the appearance of a mild cytopathic effect, infected cells were fixed in acetone for 1 h at -20°C, dried and incubated with hybridoma supernatants at 1:2 dilution in PBS for 1 h at 37°C. Cells were washed with PBS before a 30-min incubation with FITC-labeled goat anti-mouse immunoglobulin (produced in-house). The cells were washed again, stained with Evans Blue and observed using fluorescence microscopy. Cells showing a strong green fluorescent signal were recorded as positive. The uninfected cells were used as a negative control.

### ELISAs

All ELISAs were performed in 96-well Nunc Maxisoarp ELISA plates.

#### Indirect ELISAs

For hybridoma screening, ELISA plates were coated with 50 μl per well of partially purified WNV antigen (produced as previously described) at saturating concentration by incubation overnight at 4°C in ELISA coating buffer (0.05 M carbonate/bicarbonate buffer pH 9.6). Plates were washed three times with 250 μl of wash buffer (PBS containing 0.05% Tween 20) with an automatic plate washer after which 50 μl of undiluted hybridoma culture supernatants were added to each well and incubated for 1 h at 37°C. After three washes, an HRP-conjugated goat anti-mouse immunoglobulin antibody (produced in-house) was added at dilution 1/500 in dilution buffer (PBS with 0.05% Tween 20 and 1% yeast extract) to each well and incubated again for 1 h at 37°C. After a final wash cycle, 50 μl of substrate solution (orthophenylenediamine 0.5 mg/ml and 0.02% H_2_O_2 _in 50 mM phosphate citrate buffer pH 5) was added. After 10 min, the colorimetric reaction was stopped by the addition of 2 N sulfuric acid; absorbance values were read at 492 nm using an ELISA reader.

To investigate the MAbs-specificity, the reactivity of all MAbs was evaluated against TBEV, DENV1, DENV 2, DENV 3, DENV 4, YFV, JEV by indirect ELISAs.

#### Competitive binding assays for MAb epitope studies

Competition binding ELISAs were performed in WNV-coated immunoplates. Fifty microliters of unlabeled hybridoma culture supernatants were added at saturating dilution. After incubation for 15 min at 37°C, 25 μl of HRP-conjugated "competitor" MAb was added at pre-determined optimal dilution (which had given an absorbance value of 1-1.5 in a preliminary titration) without washing and incubated again for 1 h at 37°C. Follow a final wash cycle, 50 μl of substrate and stopping solution were added and absorbance values were determined as previously described. Wells without the addition of unlabeled MAb were used as negative control and wells with the addition of unlabeled MAb (the same as the HRP-conjugated MAb) were used as a positive control. Results were expressed as percent binding of HRP-conjugated MAb. The amount of binding obtained in the absence of unlabeled antibody was set at 100% for each HRP-conjugated MAb.

#### Competitive ELISA with experimental sera

Competitive ELISAs were designed to analyze the capability of known experimental horse and avian sera to inhibit the binding of anti-WNV MAbs to the antigen. Fifty microliters of known sera, at sequential dilutions starting from 1/5, were incubated for 1 h at 37°C in WNV-coated immunoplates, together with 25 μl of HRP-conjugated MAb at the pre-determined optimal dilution. After washes, the colorimetric reaction was achieved as previously described and absorbance values at 492 nm determined. The optical density (OD) of each well was converted to the percent of inhibition (PI) of MAb binding by competition with serum antibodies using the following formula: PI = [1-(OD of serum-Mab mixture/OD of Mab alone)] × 100.

#### Antigen capture-ELISA (AC-ELISA)

ELISA plates were coated with purified MAbs at a concentration of 10 μg/ml and incubated overnight at 4 C in ELISA coating buffer. Fifty microliters of WNV-infected culture supernatants (listed above) at sequential dilutions were added to each well and plates were incubated for 1 h at 37°C. After washes, 50 μl of HRP-conjugated MAb at a pre-determined dilution was added and incubated again for 1 h at 37°C, followed by a final wash cycle. Diluting buffers, washing and colorimetric reactions were the same as for the ELISAs previously described. Several combinations of trapping and conjugated MAbs were tested. Uninfected cell culture supernatants were used as a negative control, and USUV and TBEV-infected culture supernatants were used to evaluate the specificity. The cut-off OD was calculated as the mean OD of negative samples plus two standard deviations.

#### Western blotting (WB)

Western blotting was performed using a standard protocol [34]. Briefly, SDS and b mercaptoethanol denatured WNV-antigen protein were separated on 12% SDS-PAGE [35] and transferred to nitrocellulose filters [36]. MAb tissue cultures were diluted 1/5 in a phosphate saline buffer at pH 7.2 containing 1% w/v of bovine albumin and 0.05% w/w of Tween 20. MAb binding was detected by incubation with alkaline phosphatase-labeled rabbit anti-mouse IgG, and the chromogenic substrate 5-bromo-4-chloro-3-indolylphosphate-Nitro Blue

Tetrazolium (BCIP/NBT). Horse sera were diluted 1/200 in the phosphate buffer described above. Antibody binding was detected by incubation with an HRP-labeled goat anti-horse IgG and the chromogenic substrate 4-Chloronaphthol (4CN).

### Immunoperoxidase (IPMA)

IPMA was used to evaluate the reactivity profile of MAbs with all flaviviruses used in this study and previously described. Infected monolayers of Vero cells on 96-well microplates were prepared for each virus as described for IF. IPMA plates were filled with PBS with 20% of H_2_O_2_, dried and incubated with 100 μl of MAbs at 1:2 and 1.4 dilution in PBS for 1 h at 37°C. Cells were washed 3 times with PBS, after which 100 μl of HRP-conjugated goat anti-mouse IgG was added to each well and plates were incubated for 1 h at 37 C. After 3 further washings, substrate (5 mg/ml of 3-amino-9-ethylcarbazole in dimethyl sulfoxide) diluted in 0.05 M AEC buffer pH 5.2 (105 ml 0.2 M acetic acid, 395 ml 0.2 M sodium acetate, and 500 ml distilled water) was added. The colour reaction was allowed to develop for 20 minutes and the plates washed once with PBS and then examined under a light microscope. The uninfected cells were used as a negative control for each MAb dilution.

### Virus-neutralization test (VNT)

The competence of MAbs to neutralize virus infectivity was investigated by VNT carried out in 96-well microplates using two different strains of WNV in parallel (Eg101, B956), belonging respectively to lineages I and II and Vero cells. Serial twofold dilutions of MAbs (2 wells/dilution) in 25 μl of serum-free culture medium were added to each well and incubated for 1 h at 37°C with an equal volume of tissue culture fluid containing 100 TCID_50 _of WNV. Virus back titration of the working dilution of virus is included, using six wells per tenfold dilution, to confirm the validity of the test results. A volume of 50 μl of Vero cells at log5 cells/ml in medium containing 10% fetal calf serum was added to each well. After incubation for 72-96 hours at 37°C with 5% CO_2_, wells were scored for cytopathic effect and neutralizing titers were expressed as the reciprocal of the final MAb dilution required to neutralize 100% of the inoculated cultures.

### Selection of neutralization escape mutants

Neutralization escape mutants were selected by growing WNV in Vero cells in the presence of neutralizing MAbs using a modification of the method described by Holzmann et al., 1989 [37]. Essentially, serial 10-fold dilutions of WNV Eg101 (10^7 ^TCID_50_/ml) were incubated for 1 h at 37°C with an equal volume of ascites fluid containing between 1 and 8 mg/ml of neutralizing MAb. Aliquots of the virus-MAb mixture (100 μl) were adsorbed to Vero cell monolayer in 24-well plates for 1 h at 37°C, after which fresh medium was added. After incubation for 7 days at 37°C, samples of the supernatants showing CPE were screened for the presence of escape mutants by trapping ELISAs employing a non-neutralizing MAb (flavivirus-cross-reactive) as catcher and homologous neutralizing MAb in parallel with non-neutralizing MAb (flavivirus-cross-reactive) as conjugate. Mutants that were not recognized by homologous MAb while maintaining reactivity with the non-neutralizing MAb were selected and sub-cultured twice in the presence of ascites fluid. A fourth passage without MAb was performed and presumptive mutants were harvested when CPE was evident (48-72 h post-infection). Finally, each selected escape variant was cloned by limiting dilution, and amplified in Vero cells for the preparation of stock suspensions that were stored at -80°C and then titrated. The escape capability from MAb neutralization was investigated by virus neutralization tests performed using 100 TCDI_50 _of WNV selected variants and serial two-fold dilution of MAbs in Vero cells.

### Sequence analysis

Viral RNA was extracted from wild-type WNV Eg101, and WNV escape mutants using the Trizol reagent (Invitrogen, Carlsbad, CA, USA) according to the manufacturer's protocol. Regions of viral genome corresponding to genes for structural protein E were amplified for sequencing by reverse transcription-PCR [38]. One-step RT-PCR Kit (Qiagen) was used to run conventional RT-PCR assays, following the manufacturer's instructions. Amplification products were analyzed by electrophoresis on 2% agarose gels containing 0.5 μg/ml ethidium bromide. All fragments amplified by PCRs were sequenced by an automated fluorescence-based technique using an ABI-PRISM 3130 Genetic Analyzer (Applied Biosystems), following the manufacture's instruction. 3D macromolecular structure of WNV E protein was performed as described in Figure 3.

## Abbreviations

MAbs: Monoclonal antibodies; WNV: West Nile virus; USUV: Usutu virus; TBEV: Tick-borne encephalits virus; JEV: Japanese encephalitis virus; DENV1: Dengue virus serotype 1; DENV2: Dengue virus serotype 2; DENV3: Dengue virus serotype 3; DENV4: Dengue virus serotype 4; YFV: Yellow fever virus; DI: Domain I of WNV E protein; DII: Domain II of WNV E protein; DIII: Domain III of WNV E protein; IPMA: Immunoperoxidase assay; IF: Immunofluorescence; ELISA: Enzyme linked immunosorbent assay; AC-ELISA: Antigen capture ELISA; WB: Western blotting; VNT: Virus-neutralization test; CPE: Cytopathic effect; PBS: Phosphate-buffered saline pH 7.4; HRP: Horseradish Peroxidase; TCID_50_: 50% tissue culture infective dose; OD: Optical density.

## Competing interests

The authors declare that they have no competing interests.

## Authors' contributions

DL carried out most of the experiments, interpreted the results and wrote the manuscript. AM, ES, EC, IB, HZ were involved in virological and molecular analysis. EB, LC were involved in monoclonal antibodies production and in Western blotting analysis. PC designed the experiment. All of the authors have read and approved the final manuscript.
